# ImmPort, toward repurposing of open access immunological assay data for translational and clinical research

**DOI:** 10.1038/sdata.2018.15

**Published:** 2018-02-27

**Authors:** Sanchita Bhattacharya, Patrick Dunn, Cristel G. Thomas, Barry Smith, Henry Schaefer, Jieming Chen, Zicheng Hu, Kelly A. Zalocusky, Ravi D. Shankar, Shai S. Shen-Orr, Elizabeth Thomson, Jeffrey Wiser, Atul J. Butte

**Affiliations:** 1 Institute for Computational Health Sciences, University of California, San Francisco, CA 94158, USA; 2 Northrop Grumman Health Solutions, Rockville, MD 20850, USA; 3 Department of Philosophy, University at Buffalo, Buffalo, NY 14260, USA; 4 Enterprise Science and Computing Inc., Rockville, MD 20850, USA; 5 Department of Medicine, Stanford University School of Medicine, Stanford, CA 94305, USA; 6 Department of Immunology, Faculty of Medicine, Technion-Israel Institute of Technology, Haifa 3200003, Israel

**Keywords:** Immunology, Medical research, Scientific community

## Abstract

Immunology researchers are beginning to explore the possibilities of reproducibility, reuse and secondary analyses of immunology data. Open-access datasets are being applied in the validation of the methods used in the original studies, leveraging studies for meta-analysis, or generating new hypotheses. To promote these goals, the ImmPort data repository was created for the broader research community to explore the wide spectrum of clinical and basic research data and associated findings. The ImmPort ecosystem consists of four components–*Private Data, Shared Data, Data Analysis, and Resources*—for data archiving, dissemination, analyses, and reuse. To date, more than 300 studies have been made freely available through the Shared Data portal (www.immport.org/immport-open), which allows research data to be repurposed to accelerate the translation of new insights into discoveries.

## Introduction

Recent advances in high-throughput technologies, coupled with a massive accumulation of multi-scale data, has created an exciting opportunity for secondary data usage in many different areas^1,2^. ImmPort is one of the largest open repositories of subject-level human immunology data, with a commitment to promoting effective data sharing across the basic, clinical and translational research communities^3^. ImmPort collects data both from clinical and mechanistic studies on human subjects and from immunology studies on model organisms. Currently, de-identified datasets from more than 300 studies are shared through the repository with a primary focus on allergy, autoimmune diseases, infection responses, transplantation, and vaccine responses. Data and accompanying software tools are made available to the public through the ImmPort portal (http://www.immport.org/).

ImmPort is also in the vanguard of efforts to formulate and implement the standards and guidelines and demonstrate the potential of immunological assay data meta-analysis. In general, complex datasets pose challenges to data discoverability, reproducibility, and reuse. Metadata documenting data provenance can play a crucial role in overcoming these challenges^4^. The ImmPort database architecture is designed to support and maintain a variety of multi-modal immunological data such as study method documentation, metadata and standardized data formats and terminologies. Together, these efforts facilitate accurate and efficient secondary analysis of large-scale immunology data.

ImmPort was created as part of the National Institute of Allergy and Infectious Diseases Division of Allergy, Immunology and Transplantation (NIAID-DAIT) implementation of the NIH Data Sharing policy (https://grants.nih.gov/policy/sharing.htm) to promote the principles of Findability, Accessibility, Interoperability and Reusability (FAIR; https://www.force11.org/group/fairgroup/fairprinciples). In addition to collecting, curating, and sharing data from NIAID-funded clinical research projects, ImmPort now extends to embrace data from other NIH programs, including the National Institute of Arthritis and Musculoskeletal and Skin Diseases (NIAMS) and National Cancer Institute (NCI), and also from privately funded researchers and collaborations.

## Results

The goal of ImmPort is to ensure that basic and clinical research data are accessible to researchers in ways that allow effective sharing of data and knowledge. In order to facilitate this process, the ImmPort ecosystem includes four major applications: *Private Data*, *Shared Data*, *Data Analysis*, and *Resources*. Immunology research data are collected and curated in the *Private Data* application with access controlled by data providers and later published through the *Shared Data* application for open access. ImmPort Galaxy (immportgalaxy.org) is a *Data Analysis* application based on the Galaxy framework^5^ to encourage use of open source cytometry analysis tools by making command line tools available in a graphical user interface. The *Resources* application provides tutorials, customized reference datasets, and data mining and analytical tools to explore the ImmPort data model and analyze its content.

In typical consortium settings, data management practices are focused on making data available to domain experts located at multiple sites for analysis and interpretation. However, collocation of multimodal data across a consortium often leads to redundancies that complicate the iterative quality control processes inherent in research data analysis. With ImmPort, all the data collected from any given clinical trial project or investigative consortium reside in a single repository. This allows research teams to demonstrate the breadth of their activities from data reporting to deposition in ways that go beyond the limitations of a publication. It also enables external investigators to discover the content, merge across data sets, and generate and test new hypotheses and insights. Moreover, ImmPort provides a one-step registration process that ensures compliance with the key elements of the NIAID-DAIT Data Sharing Use Agreement (http://www.immport.org/agreement). As of the January 2018 data release, ImmPort shares 309 studies, 1369 experiments, 236 lab test panels, and 449 assessments (clinical, experimental or questionnaire based) from 50,180 human and animal subjects.

### Data Collection

**Figure 1:**
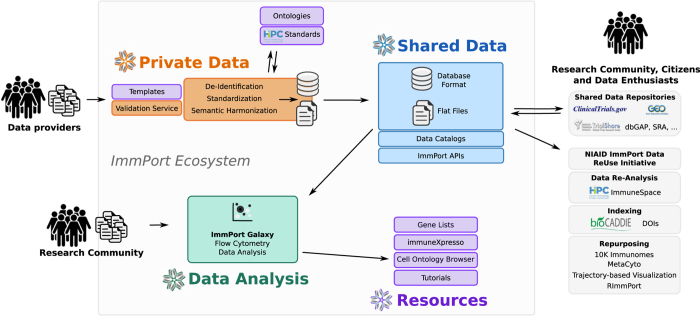
ImmPort Data Flow. The ImmPort ecosystem is composed of four applications: *Private Data, Shared Data, and Data Analysis and Resources. Private Data* is the data acquisition and curation site, *Shared Data* supports searching and distribution of data, *Data Analysis* provides a graphic interface for open source data management and analysis tools primarily focused on flow cytometry results, and *Resources* provides advanced data analysis tools, tutorials, documentation. This schematic representation shows the steps of data capture, data curation, and secondary usage of ImmPort studies by different entities. The solid line denotes an example flow of cytometry data across the four applications.

The ImmPort data collection, curation, and sharing process is the product of extensive prototyping and refinement involving DAIT Program Officers, data providers, the ImmPort data curation team, and the researchers who use the shared data. Fig. 1 shows a schematic representation of this process. For an initiative of this scale to work, it is essential that the data is provided with sufficient descriptions and content in well-defined formats that will enable effective discovery and analysis. This requirement is not always aligned with the practices and priorities of the researchers who provide the data. ImmPort customizes its engagement with data providers to meet their needs and capabilities while at the same time maximizing the quality of the data content and annotations which are provided.

**Figure 2:**
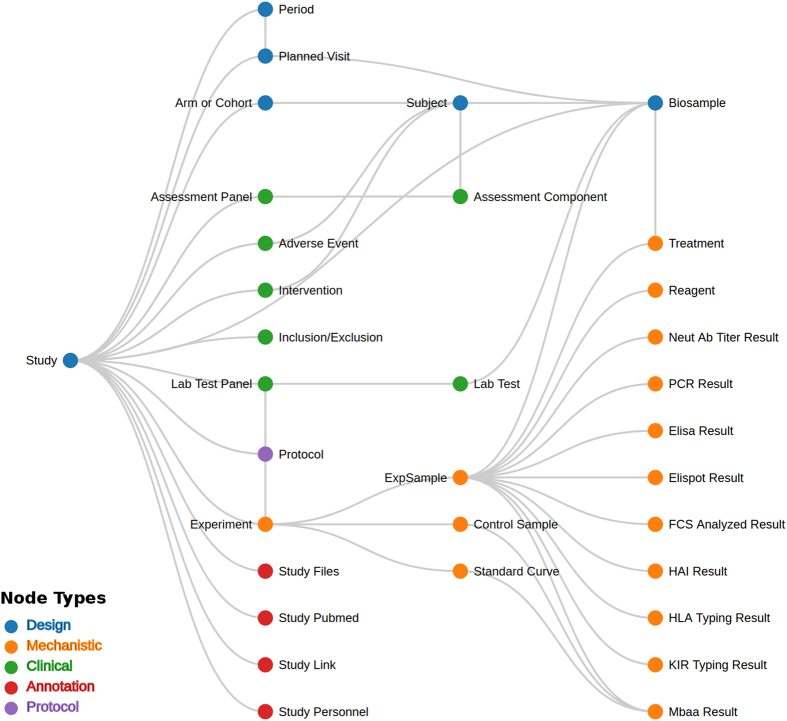
ImmPort Data Model. This figure illustrates the relationship between the nodes linking to a detailed description of the term in the ImmPort data model. The color of nodes represents the types of information stored in the ImmPort database tables.

To this end, the ImmPort has developed a set of data upload templates (http://www.immport.org/immport-open/public/home/dataTemplates) through which key elements of biomedical research data are annotated with a consistent set of descriptors. The templates are informed by community standards, and ImmPort coordinates with domain experts and standards governing bodies to upgrade and extend them to meet the needs of data providers. These templates and associated rules form the initial component of ImmPort data quality control. The data model is an aggregation of clinical and mechanistic data models from sources including Clinical Data Interchange Standards Consortium (CDISC; www.cdisc.org/), Generic Model Organism Database project (GMOD; http://gmod.org/) and Gene Expression Omnibus (GEO; https://www.ncbi.nlm.nih.gov/geo) to support immunological studies. The descriptions of studies, subjects, samples, measurements and study related metadata are captured in a relational data model (Fig. 2).

ImmPort encounters a large number of nomenclature variants across a wide spectrum of assay and assessment methods from the data providers when describing their experiments. In order to facilitate data harmonization, ImmPort has introduced standard vocabularies in the data templates to encourage data providers to use standard terms. The ImmPort data upload and curation process aims to improve the consistency with which, for example, the names of viral strains, cell surface markers, cell population definitions, lab test panels, biomarkers, assays, and assessments are displayed (see Methods).

### Human Study Participant Safeguards

ImmPort adheres to best practices in properly de-identifying human study participants. Thus, the data elements restricted by the Health Insurance Portability and Accountability Act of 1996 (HIPAA)^6^ are not captured by ImmPort (See Methods). The subject metadata includes standard demographic attributes such as age, gender, race, ethnicity and additional details, and for assay data modalities (e.g., genotyping and sequencing based assays) deemed by NIH to be potentially sensitive to re-identification, ImmPort recommends data providers upload their data to an appropriate data repository such as the Database of Genotypes and Phenotypes (dbGAP)^7^ and the Sequence Read Archive (SRA).^8^

### Clinical Trials

Clinical data sharing practices have come under increasing scrutiny with calls for improvements including the reporting of negative or contradictory results and to allow independent verification of findings on the basis of individual patient-level data (http://www.nationalacademies.org/hmd/activities/research/sharingclinicaltrialdata.aspx)^9,10^. ImmPort is the designated data sharing portal for NIAID-DAIT funded clinical trials, which include investigations into allergy, asthma, autoimmunity, infection, transplantation, and vaccine response. The ImmPort team works closely with data providers to ensure all data sets listed in case report forms, data dictionaries, publications, and study protocols are uploaded and accurately annotated. The associated clinical trials are cross-referenced with ClinicalTrials.gov and complement the latter by providing subject level results. Data from 65 clinical trials have thus far been shared through ImmPort.

### Finding and Distribution of Shared Data

**Figure 3:**
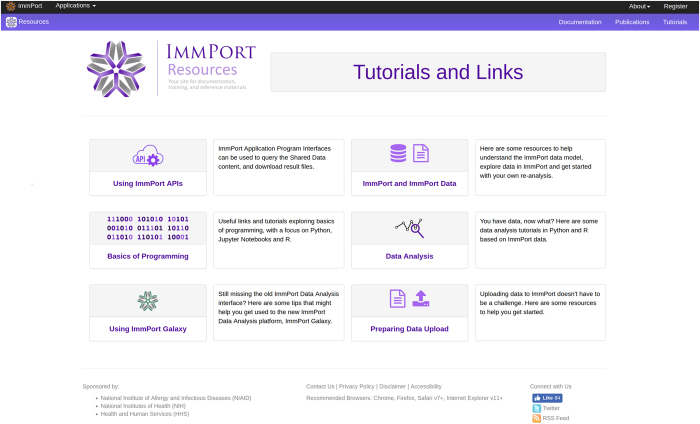
ImmPort Tutorials. The ImmPort team maintains a library of tutorials under the *Resources* application to highlight the programing tools used to explore, retrieve and analyze shared data (http://immport.org/resources/tutorials).

In recent years, ImmPort has introduced new features to facilitate fast and flexible querying across hundreds of datasets and research domains in a time-efficient manner. The *Shared Data* application incorporates a Data Catalog feature which supports searching of research data based on studies, biomarkers, experiments, assessments, and lab tests (Insert data catalog link). Subject level data is available in tab separated value (TSV) and MySQL formats. Users can choose between downloading study-specific or all shared data content. Application Programming Interfaces (APIs) are available which allow user specified extraction of data subsets based on research interest (docs.immport.org). Furthermore, ImmPort promotes educational outreach by developing tutorials which include how to use the ImmPort APIs, descriptions of the ImmPort Data Model, basics of programming and data analysis, as well as tutorials for the use of ImmPort Galaxy and data uploading to ImmPort for subsequent sharing. Concurrently, descriptive tutorials for secondary usage of data are made available for basic bench scientists in Jupyter (jupyter.org) notebooks using the R (www.R-project.org) and Python (www.python.org) programming languages (Fig. 3).

### ImmPort Galaxy for Data Analysis

**Figure 4:**
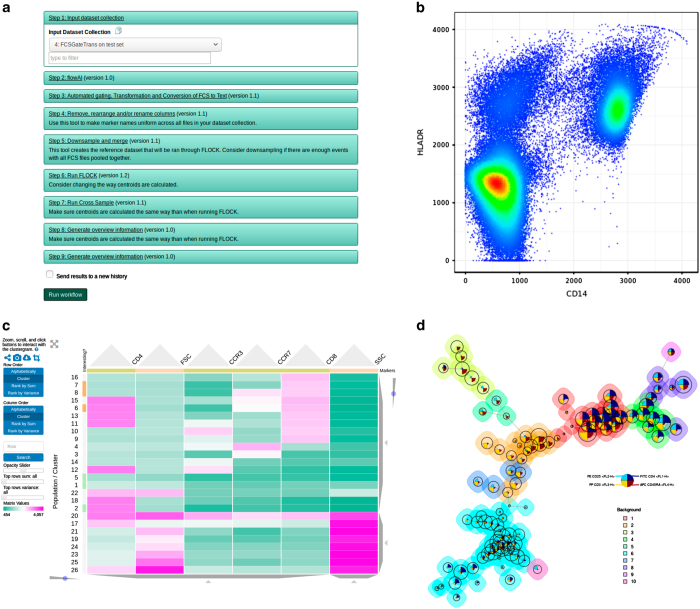
ImmPortGalaxy. The ImmPort *Data Analysis* application offers a platform to run command line analysis tools with a graphical user interface. ImmPort Galaxy supports creation and editing of workflows, tools chained together in a user-defined fashion (**a**) Available tools allow users to go from Flow Cytometry Data basic exploration (**b**) to multi-sample analysis (**c, d**).

ImmPort Galaxy is the *Data Analysis* application and seeks to promote the use of open source analysis tools applied to both conventional flow cytometry and mass cytometry (CyTOF, Cytometry Time of Flight) by providing a graphical interface for their use. This removes the barrier the standard command line interfaces pose for many immunologists^11^. ImmPort Galaxy adopts the framework established by the Galaxy Project established in making code freely available to the wider scientific community. The adaptation of the Galaxy framework to host flow analysis tools such as FLOCK^12^ and FlowSOM^13^ also addresses several data analysis challenges identified by ImmPort users in providing easier file upload, support for high-throughput analysis, and flexibility to integrate new tools (Fig. 4).

### ImmPort as a Community Resource for Data Reuse

ImmPort works with a number of consortiums to facilitate data curation and sharing between participating laboratories. The major multi-center projects includes the NIH Accelerating Medicines Partnership (AMP; https://niams.nih.gov/grants-funding/funded-research/accelerating-medicines) for Rheumatoid Arthritis (RA) and Systemic Lupus Erythematosus (SLE) seeking to define shared and disease-specific biological pathways to identify relevant drug targets for the treatment of autoimmune diseases, and the NIAID Human Immunology Project Consortium (HIPC; www.immuneprofiling.org) which characterizes human immune responses/mechanisms elicited by vaccinations, adjuvants or natural infection by capitalizing on recent advances in immune profiling technologies. ImmPort also provides curated data sets to ImmuneSpace (https://www.immunespace.org), the HIPC data analysis resource. In addition, ImmPort supports data collection for the National Cancer Institute (NCI) Oncology Model Forum (OMF; http://oncologymodels.org) and its projects to assess the quality and fidelity to human cancer of syngeneic (genetically identical) Genetically Engineered Mouse Models (GEMMs) and Patient Derived Xenografts (PDXs). In addition to government funded initiatives, ImmPort provides the March of Dimes (https://www.marchofdimes.org) Prematurity Research Centers with services to disseminate shared data resources (http://www.immport.org/resources/mod).

In the field of Immunology, the possibilities of reuse and repurposing of shared datasets are only now beginning to be explored both for validation of the methods used in their origination, but also for the creation of new knowledge through meta-analysis or through virtual testing of hypotheses not foreseen by the original authors. For example, our team reused the ANCA-Associated Vasculitis (RAVE) trial dataset in ImmPort (SDY91) to distinguish between patients who achieved remission at 6 months following Rituximab or cyclophosphamide treatment, and ‘control’ patients for whom their Rituximab treatment failed. We were able to identify distinct subsets of granulocytes as novel early markers in a subset of the patients with anti-neutrophil cytoplasmic antibody (ANCA)-associated vasculitis (AAV). This re-analysis led to novel insights and to a discovery which may better inform future clinical trials and therapies in AAV^14^.

Here, we describe three projects we are pursuing for secondary analyses. The first aims to repurpose ImmPort data to address the lack of a benchmark reference human ‘immunome’ comparable to what we have in the realm of model organism genomes. We achieve this by using all of the data relating to healthy controls obtained from the myriad of shared clinical trials and research studies within ImmPort. The result is the ‘10,000 Immunomes’ Resource^15^, a human reference data set comprising over thirteen types of measurements standardized and harmonized across the population of roughly 10,000 healthy controls, which is freely available for downloads and interactive visualizations (10kimmunomes.ucsf.edu/).

The second project takes advantage of the plethora of ImmPort shared flow and mass cytometry data across several human populations and combines them in a systematic manner. To make this possible, we developed a platform-agnostic, user-friendly, flow analysis framework called *MetaCyto*^16^ that allows cytometry data combined from multiple sources to identify demographic specific differences in circulating immune cell populations. *MetaCyto* is available as a R package on Bioconductor (www.bioconductor.org/packages/release/bioc/html/MetaCyto.html) and on the Immport Galaxy platform.

Our third project repurposes ImmPort data relating to living donors in solid organ transplantation. The curated ImmPort dataset has post-donation outcomes for kidney living donors, with a substantial subset having post-donation long-term outcome. This presents an opportunity to map the possible trajectories and sequence of events of living donors after they donate. Using such a ‘trajectory map’, we can investigate patterns of kidney donor survival and outcomes, trajectories, and the survival of kidney donors. We also foresee such an approach can be used in examining other temporal events, such as adverse drug events in clinical trial subjects. This type of data visualization tool will be made available through the *Resources* application.

In addition, we are also developing data mining and access tools to facilitate data-driven knowledge in Immunology. To date, many principles of individual cell behavior and inter-cellular circuitry have been identified. To address the resultant deluge of knowledge and to establish a foundation for systematic reasoning over the immune system inter-cellular network we built *immuneXpresso,* the first comprehensive high-resolution searchable repository of interactions between cell types and regulatory molecules identified from natural language processing of PubMed abstracts. *immuneXpresso* identifies directional relations between more than 300 cell types and 140 signaling molecules across thousands of diseases. This global high-resolution interaction map enables systematic prediction of novel cell-type-specific interactions^17^. The tool is freely accessible from the ImmPort *Resources* application (www.immport-labs.org/immport-immunexpresso/public/immunexpresso/search).

The descriptive and interpreted results data in ImmPort are structured in a relational data model. Noting that the complexity of the data model makes accessing specific data for analysis or integrating data across studies a potential challenge for researchers, we developed *RImmPort*^18^ to aggregate and format ImmPort data for analysis in the R statistical environment. To aid in the secondary use of ImmPort data, *RImmPort* implements a data model based on the CDISC clinical trial data standards, and supports a suite of functions that facilitate the data retrieval.

Furthermore, there are insightful discoveries made by other researchers from the secondary analyses of published studies from ImmPort. Khatri et al. identified a set of gene expression signatures from the blood that might help to predict the antibody responses to influenza vaccination in young individuals prior to vaccination^19^. Another data reuse example was demonstrated by a group that has retrospectively analyzed a time-series RNA-seq dataset from human peripheral blood cells^20^ to infer the distinct antibody repertoire response in individuals vaccinated with influenza vaccine^21^. Recently, a knowledge base resource was published by leveraging the gene expression and cytometry based cell phenotyping data from publicly accessible data sets (including ImmPort) as a validation set for *in silico* analyses^22^. This demonstrates the flexibility and richness of ImmPort database as a valuable data resource in Immunology.

## Discussion

ImmPort is a curation and distribution portal for promoting re-use of immunological research data generated by NIAID and other NIH programs and privately funded investigators. ImmPort provides comprehensive subject-level information from shared studies, including study design, adverse events, assessments, interventions, lab tests, medical histories, experiments and details on methods of data generation. Here we describe ImmPort’s core capabilities in making shared data Findable, Accessible, Interoperable, and Reproducible. We additionally highlight examples of reusing the clinical and mechanistic data to gain novel insights and building reference datasets and tools to benefit the community. ImmPort seeks to grow the community of shared data and open science practitioners by providing tools to search and analyze data reproducibly and tutorials to explain the data and illustrate its possibilities. The deployment of an Application Programming Interface (API) will facilitate the data exchange and interoperability within and across other resources. There is a continuing effort to engage a growing community of users which includes experimental immunologists, data-driven modelers and data enthusiasts to take advantage of the enriched ImmPort datasets deposited by large consortia and individual labs in the Immunology community.

## Method

### Sources for Standard Terms

ImmPort data is annotated with terms from several ontologies including Cell Ontology^23^, Disease Ontology (disease-ontology.org), Ontology for Biomedical Investigations (OBI; obi-ontology.org), Protein Ontology^24^, and Vaccine Ontology^25^. MedDRA (www.meddra.org) is used for adverse event terms and the NCI Thesaurus supplies terms from a variety of sources (e.g., CDISC). The Antibody Ontology (AntiO) is a new resource developed from data curated in ImmPort to provide standardized representation of monoclonal antibodies used in immunology research^26^. Along with updates to OBI, it exemplifies the ongoing development of data standardization facilitated by ImmPort. An analogous problem arises in the case of cytokines, where no public domain registry has thus far been available. To fill this gap, a registry of cytokines, chemokines and their receptors was compiled (http://www.immport.org/immport-open/public/reference/cytokineRegistry) for the purpose of collecting, integrating, and mapping between entity names and synonyms. The cytokine registry draws on resources such NCBI Gene, HGNC, MGI, Protein Ontology, and UniProt. ImmPort engages with several data standards communities such as the Human Immune Phenotyping Consortium (HIPC) Standards Working Group^18^, BioSharing (fairsharing.org), the Patient Derived Tumor Xenograft Minimal Information (PDX-MI) working group^27^ and the NIH Big Data to Knowledge (BD2K) initiative (datascience.nih.gov/bd2k/about) through its collaboration with CEDAR (http://metadatacenter.org).

### ImmPort Tools and Resources

#### MetaCyto

MetaCyto is a computational pipeline that performs automated meta-analysis of cytometry data. It allows the joint analysis of heterogeneous cytometry studies, such as studies with batch effects or studies using non-identical antibody panels. MetaCyto is available on GitHub (github.com/hzc363/MetaCyto) and on Bioconductor (http://bioconductor.org/packages/release/bioc/html/MetaCyto.html).

#### RImmPort

RImmPort is an R-driven interface to ImmPort data based on the CDISC clinical trial data standards. RImmPort supports standard data queries and analyses, and facilitates data integration across studies in ImmPort and potentially, in other repositories. The RImmPort package is released in R/Bioconductor (bioconductor.org/packages/RImmPort).

#### AntiO

AntiO provides metadata that links antibody products not only to the vendors and vendor catalog information but also to the clone that produced the antibody, their protein targets, and their fluorescent conjugations. This information is provided in an ontology form that allows for advanced querying for antibodies and their targets, so that one can easily identify, for instance, all PE-labeled anti-CD25 antibodies, their vendors and catalog numbers, and the ImmPort studies in which they were employed.

## Additional information

**How to cite this article**: Bhattacharya, S. *et al.* ImmPort, toward repurposing of open access immunological assay data for translational and clinical research. *Sci. Data* 5:180015 doi:10.1038/sdata.2018.15 (2018).

**Publisher**’**s note**: Springer Nature remains neutral with regard to jurisdictional claims in published maps and institutional affiliations.
